# Rapid Inactivation In Vitro of SARS-CoV-2 in Saliva by Black Tea and Green Tea

**DOI:** 10.3390/pathogens10060721

**Published:** 2021-06-08

**Authors:** Eriko Ohgitani, Masaharu Shin-Ya, Masaki Ichitani, Makoto Kobayashi, Takanobu Takihara, Masaya Kawamoto, Hitoshi Kinugasa, Osam Mazda

**Affiliations:** 1 Department of Immunology, Kyoto Prefectural University of Medicine, Kamigyo, Kyoto 602-8566, Japan; ohgitani@koto.kpu-m.ac.jp (E.O.); masaharu@koto.kpu-m.ac.jp (M.S.-Y.); hashibirokou8633@ezweb.ne.jp (M.K.); 2 Central Research Institute, ITO EN, Ltd., Makinohara, Shizuoka 421-0516, Japan; m-ichitani@itoen.co.jp (M.I.); m-kobayasi@itoen.co.jp (M.K.); t-takihara@itoen.co.jp (T.T.); h-kinugasa@itoen.co.jp (H.K.)

**Keywords:** novel coronavirus, COVID-19, tea, catechin, theaflavin, saliva

## Abstract

Saliva plays major roles in the human-to-human transmission of SARS-CoV-2. If the virus in saliva in SARS-CoV-2-infected individuals can be rapidly and efficiently inactivated by a beverage, the ingestion of the beverage may attenuate the spread of virus infection within a population. Recently, we reported that SARS-CoV-2 was significantly inactivated by treatment with black tea, green tea, roasted green tea and oolong tea, as well as their constituents, (-) epigallocatechin gallate (EGCG), theasinensin A (TSA), and galloylated theaflavins. However, it remains unclear to what extent tea inactivates the virus present in saliva, because saliva contains various proteins, nitrogenous products, electrolytes, and so on, which could influence the antivirus effect of tea. Here, we assessed whether tea inactivated the SARS-CoV-2 which was added in human saliva. A virus was added in healthy human saliva in vitro, and after treatment with black tea or green tea, the infectivity of the virus was evaluated by TCID_50_ assays. The virus titer fell below the detectable level or less than 1/100 after treatment with black tea or green tea for 10 s. The black tea-treated virus less remarkably replicated in cells compared with the untreated virus. These findings suggest the possibility that the ingestion of tea may inactivate SARS-CoV-2 in saliva in infected individuals, although clinical studies are required to determine the intensity and duration of the anti-viral effect of tea in saliva in humans.

## 1. Introduction

SARS-CoV-2 is transmitted from a person to another mainly through droplet infection and contact infection [1]. SARS-CoV-2 in saliva, sputum or nasal discharge is released from infected individuals by talking, sneezing, coughing, and singing, and reaches the nasopharyngeal or oral mucosa of nearby persons through inhalation, ingestion, touching using fingers, etc. [2,3,4,5,6]. In particular, virus in saliva plays important roles, because saliva is commonly excreted during talking, which is essential for our ordinary communication with each other, as well as for economic and social activities in our societies. If the SARS-CoV-2 in saliva of infected individuals is effectively inactivated by a beverage, the intake of it could attenuate the spread of virus infection within a population, while causing relatively low negative influence on economic and social activities.

Recently, we reported that SARS-CoV-2 is significantly inactivated by a treatment with green tea, roasted green tea, oolong tea, and most remarkably, black tea [7]. One min was sufficient for the inactivation of the virus by tea. As for the active constituents, (-) epigallocatechin gallate (EGCG), theaflavin-3,3′-di-gallate (TFDG) and theasinensin A (TSA) strongly inactivated SARS-CoV-2. EGCG and TFDG are bioactive compounds in green tea and black tea, respectively [8,9,10,11,12], while TSA is a dimer of EGCG [12]. We also found that TSA and TFDG significantly inhibited interaction between recombinant ACE2 and RBD of the Spike protein, which may be, at least partially, involved in the mechanisms underlying the anti-coronavirus effect of tea.

Therefore, potentially, tea could be useful for the inactivation of SARS-CoV-2 in the saliva of infected persons. However, it remains unclarified as to what extent tea could inactivate the virus present in human saliva that contains various constituents. Here, we performed an in vitro analysis to ask whether tea could inactivate the virus added in healthy human saliva.

## 2. Materials and Methods

### 2.1. Virus, Cells and Culture Medium

SARS-CoV-2 (previous nomenclature: Japan/AI/I-004/2020; present nomenclature: JPN/TY/WK-521) was generously provided from Japan National Institute of Infectious Diseases (Tokyo, Japan). VeroE6/TMPRSS2 cells [13] were obtained from Japanese Collection of Research Biosources Cell Bank, National Institute of Biomedical Innovation (Osaka, Japan) and maintained in Dulbecco’s modified Eagle’s minimum essential medium (DMEM) (Nissui Pharmaceutical Co. Ltd., Tokyo, Japan) supplemented with G418 disulfate (1 mg/mL), penicillin (100 units/mL), streptomycin (100 μg/mL), 5% fetal bovine serum at 37 °C in a 5% CO_2_/95% in a humidified atmosphere.

### 2.2. Reagents

Tea extracts were prepared by soaking 40 g of ground up and homogenized tea leaves in 2000 mL water at 80 °C for 30 min. After centrifugation at 4000 rpm for 15 min, supernatants were collected and filtrated through Toyo No. 2 filter papers, followed by evaporation and freeze-drying. Human saliva was purchased from Lee Biosolutions (Maryland Heights, MO, USA) (Appendix A Table A1).

### 2.3. TCID_50_ Assay for Virus That Was Added in Saliva and Exposed to Tea

Freeze-dried powders of green tea and black tea were dissolved in sterilized distilled water at 78 °C for 10 min to prepare x1 concentration of original tea. After chilling at room temperature, each tea was passed through a 0.45 μm filter. Human saliva was sterilized by UV irradiation for 30 min. Virus suspension (3.0 × 10^5^ TCID_50_/5 μL) was mixed with 45 μL of saliva or water, followed by an addition of tea at 1:1 (vol:vol) for 10 s. Immediately, the virus/saliva/tea mixture was diluted 1000-fold in serum-free DMEM, followed by a serial dilution at 10-fold with MS in 96-well-plates. Chilled on ice, 100 μL of each sample was added to the VeroE6/TMPRSS2 cells that had been seeded into 96-well-plates at 5 × 10^4^/100 μL/well a day before. After culture for 4 days, cells were washed, fixed and stained with crystal violet solution to estimate CPE as described [14].

### 2.4. TCID_50_ Assay to Assess Secondary Virus

Virus was added in saliva as above and mixed with equal volume of black tea, green tea or distilled water for 10 s The mixture was immediately diluted 1000-fold as above. One-hundred μL of the mixture was added to the cells that had been seeded in 24-well-plates at 2.5 × 10^5^/well a day before. After incubation for 1 h to allow attachment of the virus onto the cells, supernatant was replaced by 500 μL of fresh culture medium, and cells were cultured for 10 h. An aliquot of culture supernatant was subjected to a serial dilution at 10-fold with MS followed by TCID_50_ assay as above.

### 2.5. Calculation of TCID_50_ Values

**Figure 1:**
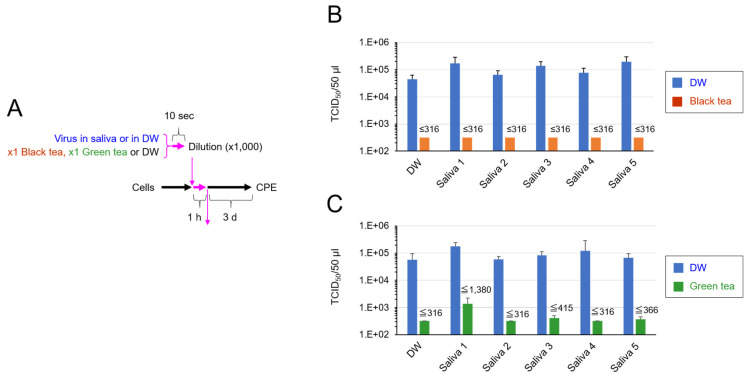
SARS-CoV-2 was inactivated by tea in the presence of saliva. SARS-CoV-2 was diluted in saliva from five independent donors or in distilled water (DW) as a control. Black tea, green tea or DW was added to the virus/saliva for 10 s, immediately followed by a 1000-fold dilution with MS. TCID_50_ assay was performed as described in the Materials and Methods. Scheme of experiment (**A**) and virus titer of each sample (means ± S.D., *N* = 3) (**B**) are shown, (**C**) Green tea also significantly declined the titer of the virus in saliva.

**Figure 2:**
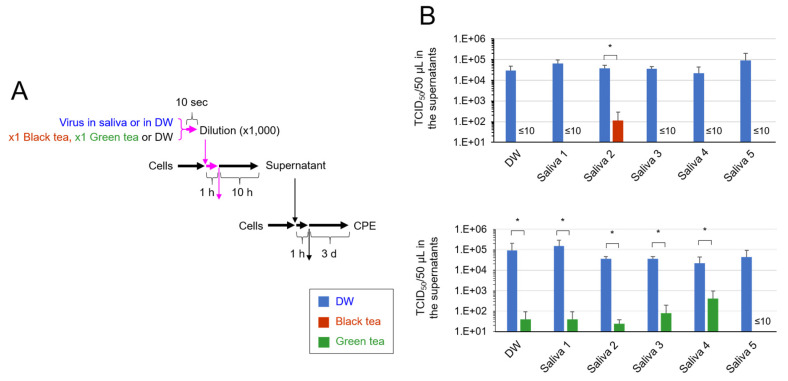
Treatment with tea significantly prevented generation of secondary virus. SARS-CoV-2 was diluted in saliva from five donors or in DW. Black tea, green tea or DW was added to the virus/saliva for 10 s, immediately followed by a 1000-fold dilution with MS. After infection, cells were cultured for 10 h, and culture supernatants were harvested. After serial dilution, the supernatants were infected to cells to determine TCID_50_ values. Scheme of experiment (**A**) and virus titer of each sample (means ± S.D., *N* = 3) (**B**) are shown. * *p* < 0.05, between groups.

TCID_50_ values were calculated by the Reed–Muench method as described elsewhere. In some TCID_50_ assays, virus suspension was diluted 1000-fold before serial dilution as described. Therefore, the detectable limits in these TCID_50_ assays (Figure 1) were higher than those in other assays (Figure 2). If one or more triplicate wells of the lowest dilution of a sample did not show CPE, the highest possible average of TCID_50_ value was calculated for the sample.

### 2.6. Real Time-RT-PCR Analysis

Virus added in saliva was mixed with equal volume of black tea or distilled water for 10 s, followed by an immediate dilution 1000-fold as above. One hundred μL of the mixture was added to the cells in 24-well-plates, and 1 h later supernatant was replaced by 500 μL of fresh culture medium. After culture for 10 h, RNA was extracted from culture supernatant and from cells using TRI Reagent^®^ LS (Molecular Research Center, Inc., Montgomery Road, Cincinnati, OH, USA). After reverse-transcription using ReverTra Ace^®^ qPCR RT Master Mix (Toyobo, Shiga, Japan), cDNA was subjected to real-time PCR using a Step-One Plus Real-Time PCR system (Applied Biosystems, Foster City, CA, USA) and the following primers/probes specific for viral N gene: Forward primer, 5′-AAATTTTGGGGACCAGGAAC-3′; reverse primer, 5′-TGG-CAGCTGTGTAGGTCAAC-3′; and probe, 5′-(FAM) ATGTCGCGCATTGGCATGGA (BHQ)-3′.

### 2.7. Statistical Analysis

Statistical significance was analyzed by Student’s *t* test, and *p* < 0.05 was considered significant.

## 3. Results

To simulate what happens in the oral cavity of a SARS-CoV-2-infected person who ingests tea, our in vitro experiments were designed in such a way that (i) virus was added in saliva, (ii) the virus/saliva was mixed with equal volume of ×1 concentration of black tea or green tea, and (iii) the exposure time was 10 s (Figure 1A). Saliva from five different healthy donors (purchased from Lee Biosolutions) (Appendix A Table A1) was used. As shown in Figure 1B, the infectivity of the black tea-treated virus dropped below detection level or less than 1/100 of untreated virus, even in the presence of the saliva. Green tea also significantly declined the titer of the virus in saliva (Figure 1C).

To further confirm the inactivation of virus in saliva by tea, virus added in saliva was exposed to black tea or green tea for 10 s before infection with cells, and after culture, secondary virus generated from the cells was estimated by TCID_50_ assay (Figure 2A). As shown in Figure 2B, virus titers in culture supernatants were either not detected or significantly lower compared with the titer of secondary virus released from the cells infected with intact virus. The results strongly suggest that the SARS-CoV-2 added in saliva and treated with black tea or green tea had quite low capability of infection.

**Figure 3:**
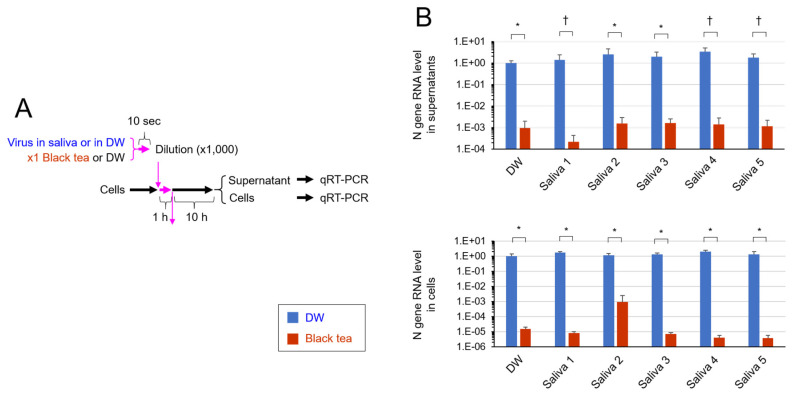
Treatment with tea significantly inhibited replication of SARS-CoV-2 in cells. SARS-CoV-2 was diluted in saliva from five donors or in DW. Black tea or DW was added to the virus/saliva for 10 s, immediately followed by a 1000-fold dilution with MS. After infection, cells were cultured for 10 h. RNA was extracted from the culture supernatants and from cells, and real time-RT-PCR was performed to evaluate viral N gene RNA, as described in Materials and Methods. Scheme of experiment (**A**) and means ± S.D. of relative RNA levels (*N* = 3) (**B**) are shown. * *p* < 0.05, between groups. ^†^ *p* values cannot be calculated because one or two of the triplicate data was “0”.

RNA was extracted from the cells as well as from the culture supernatant to evaluate viral N gene RNA by quantitative RT-PCR (Figure 3A). Culture supernatant of the cells that were infected with black tea-treated SARS-CoV-2 contained remarkably fewer copies of N gene RNA compared with the supernatant of cells infected with untreated virus (Figure 3B, Upper). This is consistent with the low virus titer in the supernatant mentioned above (Figure 2B, Upper). Viral RNA level was also lower in the cells that were infected with black tea-treated SARS-CoV-2 than in the cells infected with nontreated virus (Figure 3B, Lower), strongly suggesting the low capability of black tea-treated virus to infect and replicate in cells.

## 4. Discussion

Saliva is the major origin of droplets that are released from SARS-CoV-2-infected persons and cause droplet infection and contact infection to other persons [3,4,5,6,15]. Saliva contains various proteins, including mucins, enzymes and immunoglobulins, nitrogenous products such as urea and ammonia, and electrolytes [16]. The present study shows that tea significantly inactivates SARS-CoV-2 even in saliva (Figure 1, Figure 2 and Figure 3).

In our experiments, the virus was first added to human saliva, followed by a treatment with the same volume of tea for 10 s (Figure 1, Figure 2 and Figure 3). The virus was remarkably inactivated by such treatment (Figure 1). The tea-treated virus replicated less vigorously in infected cells (Figure 3) and produced lower titers of secondary virus from the cells (Figure 2), compared with the untreated virus. These data suggest the possibility that tea ingestion/rinsing may result in inactivation of SARS-CoV-2 in saliva in infected persons. Similar results were obtained using saliva from five different donors (Figure 1, Figure 2 and Figure 3), but it should be important to examine saliva from a larger number of donors in the future.

When a noninfected person ingests tea before or after close contact with another person who potentially could be an asymptomatic carrier of SARS-CoV-2, tea may prevent infection through the oral route to some extent. Tea should be placed in the oral cavity with mouth closed and swished throughout the oral cavity for more than 10 s before swallowing. However, tea may not effectively prevent viral infection via nasal and respiratory mucosa. Thus, the prophylactic effect of tea may be limited.

When a SARS-CoV-2-infected person ingests tea, it may inactivate the virus in oral cavity, oro- and laryngo-pharynx and, potentially, gastrointestinal tract. Infection at the GI tract is regarded as an important characteristic of COVID-19 [17,18,19]. However, oral ingestion of tea may not be effective for the treatment of pulmonary and systemic manifestations of COVID-19 patients, because catechins and theaflavins that are active constituents for the anti-SARS-CoV-2 effect [7] are poorly absorbed into circulation [20,21,22,23]. It remains unclear whether EGCG, TSA and TFDG can be safely and effectively administered to patients through intravenous, intradermal, intranasal or inhalation routes. Thus, the therapeutic effect of tea may also be limited.

More importantly, present findings suggest the utilization of tea in a novel “public health intervention”-like strategy to combat COVID-19. If a lot of people (among which some asymptomatic carriers could be present) ingest a small amount of tea before having close contact with other persons, going to a restaurant, hospital, or crowded place, etc., for a couple of weeks during a period of explosive surge of COVID-19, such activities could potentially attenuate the transmission of infection to each other [24]. Besides, tea gargling may be recommended to patients who undergo dental treatment, upper GI endoscopic examination, and so on, to prevent nosocomial infection of SARS-CoV-2, in case some of the patients could be asymptomatic carriers of SARS-CoV-2.

The in vivo effect of tea ingestion may be transient, because the newly generated virus should reaccumulate over time in the saliva in SARS-CoV-2 infected persons after the tea ingestion. Turnover in vivo of the virus in saliva has not been reported so far, except for a study by Yoon et al., who examined a time-course of change of viral load in saliva in COVID-19 patients after mouthwash with chlorhexidine [25]. They reported that the viral load dropped 1 h after the disinfectant mouthwash in two patients. The viral load remained undetectable for 2 h, and jumped up at 3 h, in the patients suggesting reaccumulation of newly generated virus between 2 and 3 h after transient elimination of preexisting virus by the mouthwash. Clinical studies in the future may reveal whether the virus reaccumulates in a similar time-course after inactivation of SARS-CoV-2 by tea in saliva in infected individuals.

Again, therapeutic and prophylactic efficacies of tea ingestion may not be very promising. Tea ingestion may be effective to some extent to attenuate spread of SARS-CoV-2, if it is used in a “public health intervention”-like procedure, as above. Clinical studies are needed to assess potential efficiency of such a procedure. Tea ingestion may not inactivate SARS-CoV-2 in nasal discharge and airway mucosal fluid. Thus, respiratory aerosol-mediated infection may not be prevented by tea ingestion. Although evidence has not been presented for respiratory aerosol-mediated infection, caution is needed for it [26].

## Data Availability

Not applicable.

## Sample Availability

Samples are not available from the authors.

## Appendix A

**Table A1:** Donors of saliva that was purchased from Lee Biosolutions (Maryland Heights, MO, USA) and used in the study are shown.

Number	Birth Year	Gender	Race
1	1984	Male	Caucasian
2	1989	Male	African American
3	1986	Male	Caucasian
4	1967	Female	Caucasian
5	1968	Male	Caucasian
